# 160 GHz D-Band Low-Noise Amplifier and Power Amplifier for Radar-Based Contactless Vital-Signs-Monitoring Systems

**DOI:** 10.3390/mi14050993

**Published:** 2023-05-02

**Authors:** Ademola Akeem Mustapha, Mihai Sanduleanu

**Affiliations:** 1 Electrical Engineering and Computer Science Department, Khalifa University of Science and Technology, Abu Dhabi P.O. Box 127788, United Arab Emirates; mihai.sanduleanu@ku.ac.ae; 2 System on Chip Center, Khalifa University, Abu Dhabi P.O. Box 127788, United Arab Emirates

**Keywords:** amplifier, cascode, common source, contactless monitoring, D-band, low-noise amplifier, power amplifier, radar, vital signs

## Abstract

This paper presents a 160 GHz, D-band, low-noise amplifier (LNA) and a D-band power amplifier (PA) implemented in the Global Foundries 22 nm CMOS FDSOI. The two designs are used for the contactless monitoring of vital signs in the D-band. The LNA is based on multiple stages of a cascode amplifier topology with a common source topology adopted as the input and output stages. The input stage of the LNA is designed for simultaneous input and output matching, while the inter-stage-matching networks are designed for maximizing the voltage swing. The LNA achieved a maximum gain of 17 dB at 163 GHz. The input return loss was quite poor in the 157–166 GHz frequency band. The −3 dB gain bandwidth corresponded to 157–166 GHz. The measured noise figure was between 7.6 dB and 8 dB within the −3 dB gain bandwidth. The power amplifier achieved an output 1 dB compression point of 6.8 dBm at 159.75 GHz. The measured power consumptions of the LNA and the PA were 28.8 mW and 10.8 mW, respectively.

## 1. Introduction

The regular monitoring of vital signs, especially heart rate (HR) and respiratory rate (RR), is important for the early detection of abnormal health conditions that could lead to critical health problems [1]. Radar-based vital-signs-monitoring systems consist of transceiver circuits for transmitting and receiving signals to and from patients. Some of the key components of the transceiver circuit include a low-noise amplifier and a power amplifier [2,3]. Due to the relatively low signal level of the transmitted and received signals, focusing on the implementation of the low-noise amplifier of the receiver path is necessary. Similarly, for the transmitter path, an efficient power amplifier is sought to achieve this task.

High-performance low-noise amplifiers are essential for high-sensitivity receivers as they determine the receiver noise figure. Common-source amplifiers with source degeneration are commonly used at low frequencies (0.1–40 GHz) to achieve both gain and noise matching. However, the degenerated inductor used in this type of amplifier can reduce its gain, which is only acceptable at these frequencies due to the high intrinsic transistor gain. As the frequency increases, such as in the V-band, E-band, and W-band, the common-source amplifier is often replaced by a cascode amplifier, which provides higher gain per stage [4,5,6,7,8,9,10,11,12,13,14].

On the other hand, power amplifiers (PAs) operating in the millimeter-wave frequency band are difficult to design and implement due to the need for high gain, output power, and linearity and a wide bandwidth. The speed limitations of CMOS devices make it challenging to implement sub-millimeter-wave PAs. Several techniques have been proposed to address these challenges, including capacitive neutralization [15,16], current-combining transformers [17], direct combining, pulse injection [18], and Doherty topology [19]. 

This paper proposes a D-band LNA and PA for vital-signs-monitoring systems. The high-frequency band is preferred because of the low form factor of the antenna and the improved spatial resolutions at this frequency band. A multistage design approach is employed in order to achieve high gain. Unlike most reported designs in this frequency band, common-source amplifiers are employed for the input and output stages of the LNA for simultaneous gain and noise matching. However, cascode amplifiers are used for the inter-stage amplifiers. The PA is based on a class-A topology.

The paper is organized as follows: Section 2 discusses the vital-signs-monitoring system architecture for the proposed LNA and PA. Section 3 provides details on the circuit’s implementation. Measurement results are presented in Section 4, while conclusions are drawn in Section 5.

## 2. System Architecture

**Figure 1:**
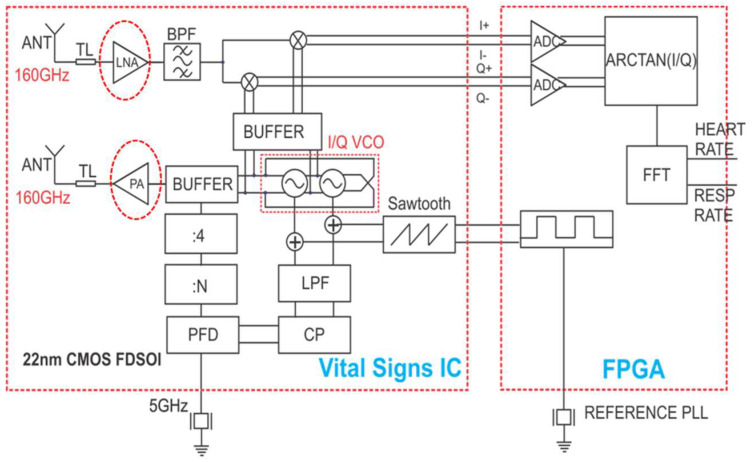
The LNA and PA are constitutive parts of a vital-signs-monitoring IC with integrated antennas operating at 160 GHz.

The PA and the LNA are proposed for the system architecture shown in Figure 1. The transmitter (Tx) and receiver (Rx) antennas are integrated on-chip, and the Rx and Tx share the same integer-N PLL with I/Q VCO. At the Tx side, the output signal from the VCO is applied to a buffer, and the buffer’s output is connected to the input of the PA. At the Rx side, the received antenna signal is amplified by the LNA, bandpass-filtered, and then demodulated using the I/Q mixer. The baseband signals are digitized by the ADC and processed by an FPGA to obtain the HR and RR.

## 3. Circuit Design

**Figure 2:**
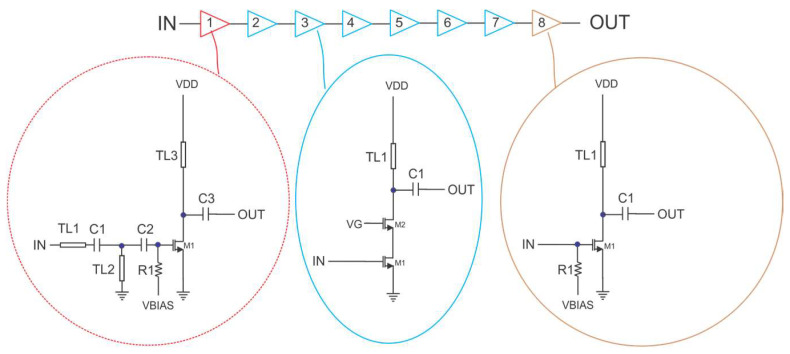
The low-noise amplifier’s circuit diagram.

Figure 2 shows a schematic diagram of the LNA. The LNA is designed as a single-ended, eight-stage, cascaded amplifier with a mix of common source and cascode topologies. The first stage is the common-source (CS) amplifier stage. In this paper, the choice of the CS stage instead of the cascode stage is related to noise. Although a cascode stage improves the S_12_, the cascode transistor introduces extra noise at higher frequencies, which manifests at frequencies greater than (1).

(1) $$f=\frac{1}{2r_{01}C_{x}}$$

In Equation (1), $r_{01}$ is the output resistance of the CS transistor in the cascode amplifier stage and $C_{x}$ is the parasitic capacitance at the source of the cascode transistor.

As in the first stage, the last stage is also implemented as a CS stage. However, stages 2, 4, 5, 6, and 7 are implemented as cascode stages to minimize the S_12_ of the LNA. The noise of these stages is less critical, as the first stage’s gain will render the noise insignificant.

A T-matching topology (C1, TL2, and C2) was adopted at the input for noise and impedance matching. TL2 is an inductor implemented with a shorted transmission line. TL1 accounts for the routing distance from capacitor C1 to the input pad. Capacitor C1 acts as a DC-blocking capacitor, as the shunt transmission line TL2 is connected to the ground. The input impedance at the gate of M1 is mostly capacitive. The role of TL2 is to adjust the input impedance seen at the gate of transistor M1 of the first stage to 50 Ω for matching purposes. The capacitor C2 is a DC-blocking capacitor for the bias voltage, V_BIAS_, at the gate of M1. Both C1 and C2 form part of the matching circuit. The V_BIAS_ is generated from a diode-connected transistor biased externally from a constant current source. Thus, the design is less sensitive to V_T_ mismatch as the current in each of the stages is constant. 

The signal amplified by the first stage is applied to the second stage (cascade stage) through a decoupling capacitor (C3), whose value is not important as long as its value is large enough. The second amplifier is biased through the same large-value resistor connected to the same gate-drain terminal of the diode-connected transistor from the current mirror. As a result, the DC current in all the amplifier stages is the same. The gate of the cascode transistor is connected to a constant bias voltage VG decoupled to ground, thereby ensuring that any residual RF signal is shunted to ground. The last amplification stage is identical to the first stage, and the same design considerations are valid.

Each stage uses a transmission line as a load that resonates with the capacitance observed at the input of the next stage at the desired frequency. At the output, the output impedance of the transistor, the load transmission line, and the series capacitor provide 50 Ω of impedance for matching with the output measurement equipment.

**Figure 3:**
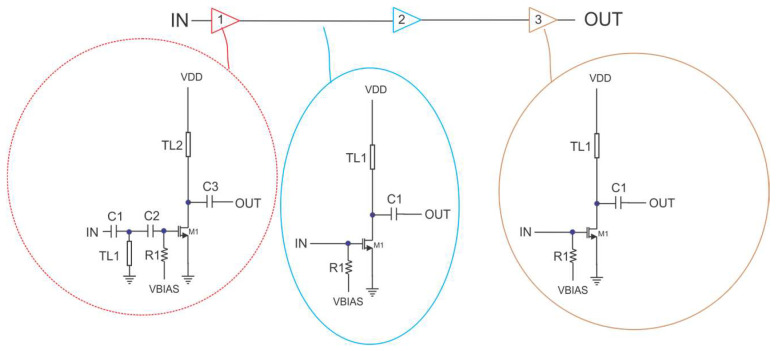
The power amplifier’s circuit diagram.

The power amplifier shown in Figure 3 consists of three CS stages with transmission line loads. The RF signal is applied through a decoupling capacitor (C1) to the input-matching circuit consisting of the transmission line TL1 and the capacitor C2. The gate of the CS amplifier is biased through a large resistor (R1). The resistor is connected to the gate of a diode-connected transistor in a current mirror configuration. The bias current in all transistors (M1) is constant and controlled from an outside current source. This type of biasing ensures less variability in the PA parameters due to VT mismatch. The signal amplified by the first stage is applied to the second stage through a decoupling capacitor (C3), whose value is not important as long it is large enough. There is no interstage matching as the PA stages are not realized in a discrete form and the stages are very close to each other. The design criteria for this purpose consist of the maximization of the voltage at the gate of the next stage. The second amplifier is biased through the same large-value resistor connected to the same gate-drain terminal of the diode-connected transistor from the current mirror. As a result, the DC current in all the amplifier stages is the same. A decoupling capacitor connected to the ground ensures that any residual RF signal is shunted to the ground. The last amplification stage is identical to the second stage, and the same design considerations are valid.

For both the LNA and PA, each transistor has a length of 22 nm and a width varying from 150–200 µm, depending on the stage. The number of fingers is around 125–166 to ensure a unit cell of 1.2 µm. This choice ensures a maximum $f_{t}$ of 300 GHz and a small gate resistance for the LNA to improve its noise figure.

## 4. Measurement Results

**Figure 4:**
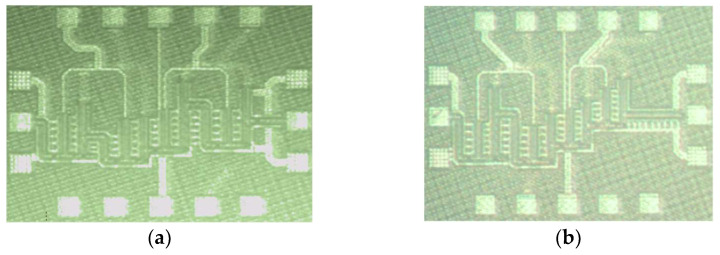
(**a**) A photomicrograph of the low-noise amplifier chip (0.65 mm × 0.45 mm); (**b**) a photomicrograph of the power amplifier chip (0.65 mm × 0.45 mm).

The LNA chip shown in Figure 4a was realized in the GF 22 nm CMOS FDSOI process and occupies an area of 0.65 mm × 0.45 mm. The PA chip shown in Figure 4b was also realized in the GF 22 nm CMOS FDSOI process and has an area of 0.65 mm × 0.45 mm.

**Figure 5:**
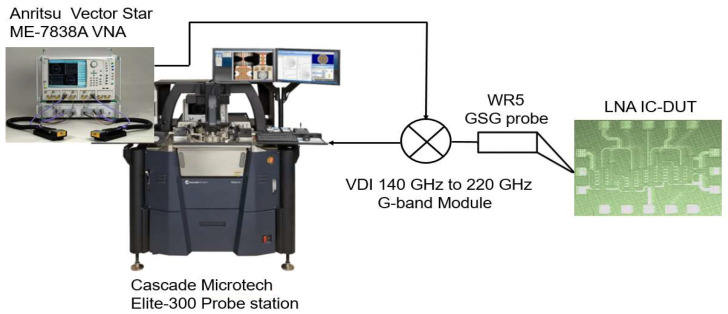
LNA measurement setup.

To measure the D-band LNA, the measurement setup from Figure 5 was used. The device under test (DUT) containing the LNA was placed on the Elite 300 Cascade Microtech probe station. The probes were used to gently make contact with the pads of the LNA DUT. The signals from the probes were applied to the 140–220 GHz G-band VDI module operating as an up-conversion mixer at the LNA input and a down-conversion mixer at the output. The converted signals were then connected to the Anritsu Vector Star ME-7838A VNA. The S-parameters and the NF were measured with the same VNA.

**Figure 6:**
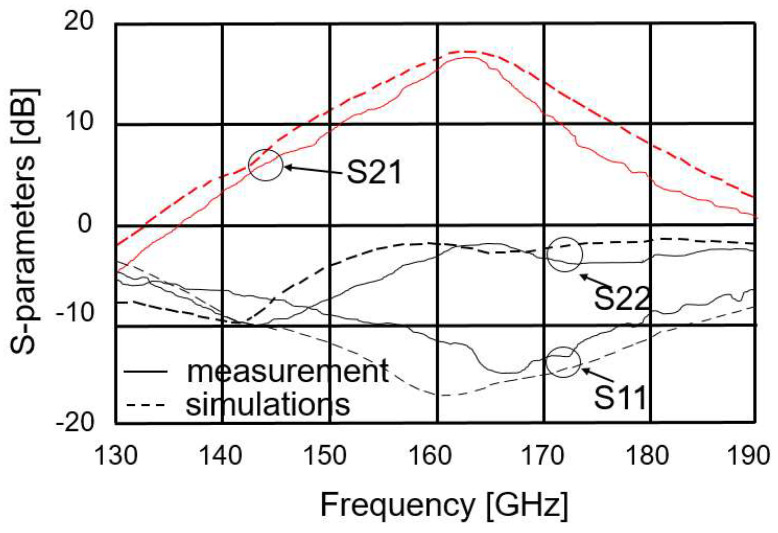
Simulated and measured S parameters of the LNA.

**Figure 7:**
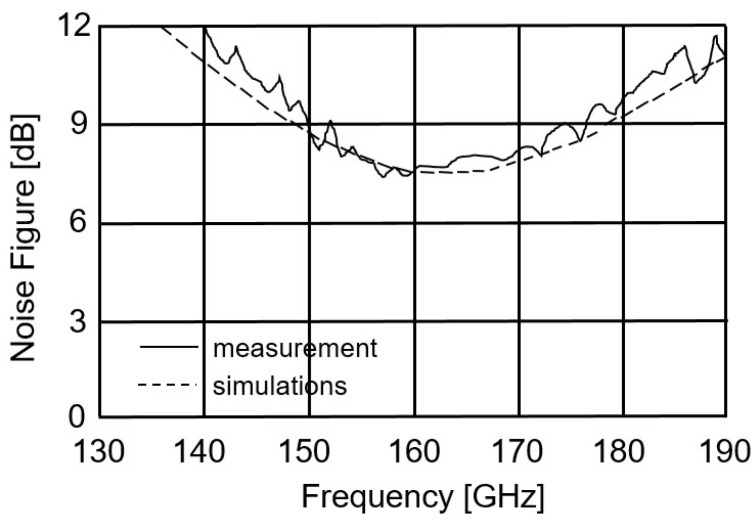
Simulated and measured noise figures of the LNA.

The measured and simulated S parameters of the LNA are presented in Figure 6. The small discrepancy between the measured and simulated results is attributed to the inaccuracy of the transistor model at 160 GHz. The power gain of the LNA (S_21_) is greater than 17 dB. The input return loss (S_11_) is greater than −10 dB at 160 GHz. The output return loss (S_22_) is not very good, but this does not affect the operation of the LNA. The measured NF of the LNA (see Figure 7) is less than 8 dB. 

**Figure 8:**
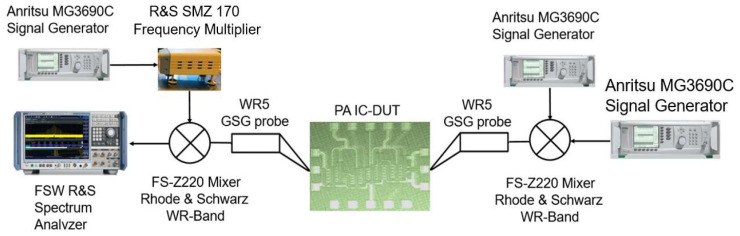
PA measurement setup.

In order to measure the D-band PA, the measurement setup of Figure 8 was used. The input of the PA DUT was accessed through a GSG probe connected to a Rhode & Schwarz FS-Z220 Mixer. The signal from the Anritsu MG3690C signal generator was applied to the IF port of the mixer, while the signal from the Anritsu MG3690C signal generator was applied to the LO port of the mixer.

**Figure 9:**
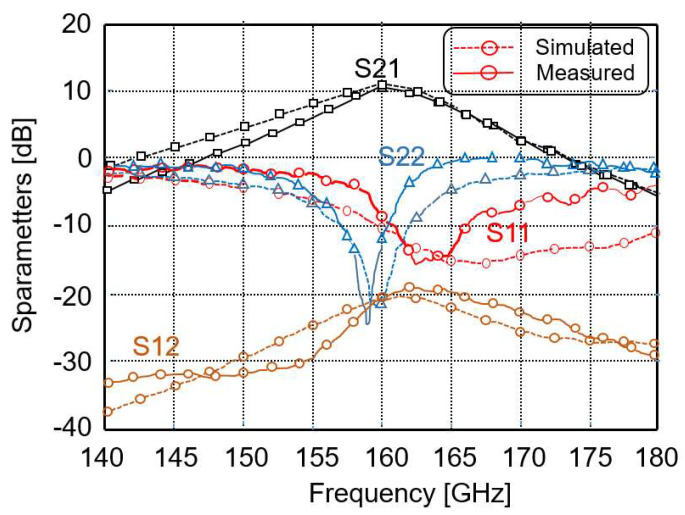
Simulated and measured S-parameters of the PA.

The measured and simulated S parameters of the PA are presented in Figure 9. The measured degree of input matching shows better matching around 162 GHz (S_11_ < −15 dB) but a smaller −10 dB bandwidth than the simulations. The measured PA power gain is greater than 10 dB at 160 GHz, and output power matching is greater than −20 dB at 157 GHz. At 160 GHz, S_22_ is equal to −11 dB, and the −10 dB bandwidth is from 156 GHz to 162 GHz.

**Figure 10:**
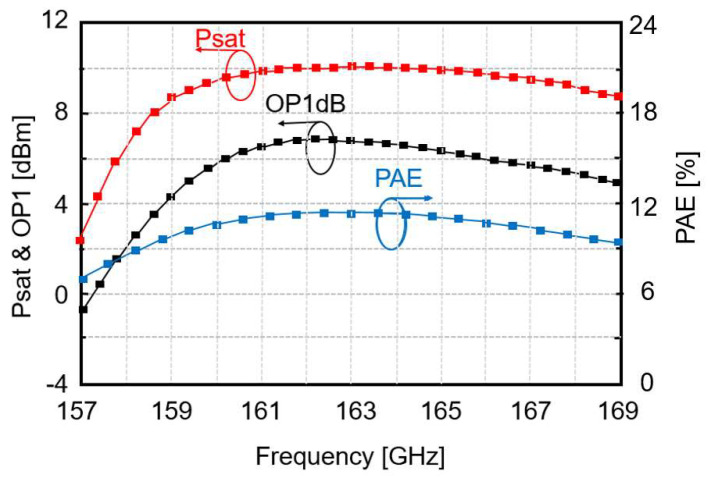
Measured *Psat*, *PAE*, and OP_1dB_ of the PA.

The large signal measurement results of the power amplifier are shown in Figure 10. The saturated output power (*P_sat_*), the output at a 1 dB compression point (OP_1dB_), and the power-added efficiency (*PAE*) are plotted against the operating frequency. The saturated output power *P_sat_* is greater than 10 dBm, whereas the output 1 dB compression point is greater than 7 dBm. The power added efficiency is greater than 11%.

For benchmarking and comparison with other sub mm Wave designs, refer to Table 1 and Table 2. Compared to other reported works in [13,14,20,21,22,23], the low-noise amplifier achieved state-of-the-art performance. According to Table 1, the proposed LNA achieves high gain, offers the second lowest amount of power consumption, and provides a low-noise figure. For a better comparison, we used the following FOM proposed in [24]:

(2) $$\mathrm{FOM}=\frac{\left(NF_{min}-1\right)P_{D}{}^{\frac{1}{3}}}{L^{\frac{4}{3}}f_{0}{}^{\frac{2}{3}}}$$

where $NF_{min}$ is the minimum noise figure, $P_{D}$ is the dissipated power (in mW), L is the technology minimum *L* (in nm), and $f_{0}$ is the center frequency (in GHz). This shows that our design has the second best FOM, although the performance presented in [23] is only based on simulated results.

Similarly, the proposed power amplifier has a relatively high power-added efficiency given the frequency range [16,25,26,27,28,29]. Its drain efficiency is 46%. It also has low power consumption, with a saturated output power of 10 dBm. Its Power-Added Efficiency (*PAE*) is much lower than its drain efficiency because the PA operates at 160 GHz. At higher frequencies, the *PAE* usually decreases as the same topology will lead to higher *PAE* at lower frequencies. Additionally, the lower *PAE* obtained is partly due to the lower gain. The *PAE* is, however, higher than the *PAE* of the designs in [16,25,26] because the topologies proposed in these references use a larger number of stages that provide higher gain but also higher power dissipation. Furthermore, the topologies use transformers that experience about 4–5 dB insertion loss at those frequencies. Some references also use a differential approach that increases power dissipation.

For a general comparison with the state of the art, we used the figure of merit proposed for the ITRS technology roadmap and expressed as (3)

(3) $$\mathrm{FOM}=P_{sat}\left(\mathrm{dBm}\right)+Gain\left(\mathrm{dB}\right)+10\log_{10}\left(PAE\right)+20\log_{10}\frac{f_{0}}{f_{max}}$$

where $P_{sat}$ is the saturated output power, *Gain* is the PA power gain, *PAE* is the power-added efficiency, $f_{0}$ is the operating frequency, and $f_{max}$ is the technology figure of merit. Based on the FOM, our design offers better performance than other designs.

## 5. Conclusions

This paper proposes D-band, 160 GHz LNA and PA implemented in the Global Foundries 22 nm FD-SOI CMOS process. The power gain of the LNA is greater than 17 dB, the input return loss (S11) is greater than −10 dB at 160 GHz, and the NF is less than 8 dB. It has a power consumption values 28.8 mW and an area of 0.29 mm^2^. The PA achieved a power gain of 10 dB, an output 1 dB compression point of 6.8 dBm, and a *PAE* of 11%. Its power consumption is 10.8 mW, and its occupied area is 0.29 mm^2^.

## Figures and Tables

**Table 1:** Comparison with existing D-band low-noise amplifiers.

Ref.	Tech.	Freq. (GHz)	*Gain* (dB)	BW (GHz)	NF (dB)	P_dc_ (mW)	Area (mm^2^)	FOM $\frac{\left(NF_{min}-1\right)P_{D}{}^{\frac{1}{3}}}{L^{\frac{4}{3}}f_{0}{}^{\frac{2}{3}}}$
[13]	FDSOI 28 nm	143–166	15.7	23	8.5	32	0.34 (core)	0.053
[14]	45 nm CMOS SOI	125.5–157	16	31.5	8	75	0.07 (core)	0.035
[20]	28 nm FDSOI	117–143	14.5	26	-	21.6	0.1 (core)	0.045
[21]	22 nm FDSOI	131–162	21	31	5.5 *	28–46	0.02 (core)	0.091
[22]	22 nm FDSOI	130	17	24.8	8	60	0.133 (core)	0.018
[23]	22 nm FDSOI	146.6–157.4	9–18	10.8	7.5–9.3 *	17.5–27.5	0.09	0.014
This work	22 nm FDSOI	157–166	17	9	7.6	28.8	0.293 (total)	0.061

* Simulated values.

**Table 2:** Comparison with existing D-band power amplifiers.

Ref.	Tech.	Freq. (GHz)	*Gain* (dB)	*P_sat_* (dBm)	OP_1dB_ (dBm)	P_dc_ (mW)	*PAE* (%)	FOM
[19]	45 nm CMOS	152.5	18	8.8	5	92	6.8	29.6
[25]	40 nm CMOS	140	20.3	14.8	10.7	305	8.9	39.49
[26]	40 nm bulk CMOS	132	22.5	8	5.2	305	6.6	37.57
[27]	22 nm FDSOI	135	14.2	10.3	9.6	30	7.9	26.53
[28]	22 nm FDSOI	130	13.5	17.5	-	56	16.5	35.90
[29]	22 nm FDSOI	135.8	23.4	6.5	4	46	9.1	32.60
This work	22 nm FDSOI	160	10	10	7	10.8	11	40.82

## Data Availability

Data sharing is not applicable.
